# Lactococcosis a Re-Emerging Disease in Aquaculture: Disease Significant and Phytotherapy

**DOI:** 10.3390/vetsci8090181

**Published:** 2021-09-03

**Authors:** Mehdi Soltani, Bernardo Baldisserotto, Seyed Pezhman Hosseini Shekarabi, Shafigh Shafiei, Masoumeh Bashiri

**Affiliations:** 1 Freshwater Fish Group and Fish Health Unit, Centre for Sustainable Aquatic Ecosystems, Harry Butler Institute, Murdoch University, Murdoch, Perth, WA 6150, Australia; 2 Department of Aquatic Animal Health, Faculty of Veterinary Medicine, University of Tehran, Tehran 1419963111, Iran; m.bashiri1369@gmail.com; 3 Department of Physiology and Pharmacology, Federal University of Santa Maria, Santa Maria 97105-900, RS, Brazil; bbaldisserotto@hotmail.com; 4 Department of Fisheries, Science and Research Branch, Islamic Azad University, Tehran 1468763785, Iran; hosseini.pezhman@yahoo.com; 5 Department of Food Hygiene and Quality Control, Faculty of Veterinary Medicine, Shahrekord University, Shahrekord 64165478, Iran; shafiei.shafigh@gmail.com

**Keywords:** lactococcosis, *Lactococcus garvieae*, phytotherapy, aquaculture

## Abstract

Lactococcosis, particularly that caused by *Lactococcus garvieae*, is a major re-emerging bacterial disease seriously affecting the sustainability of aquaculture industry. Medicinal herbs and plants do not have very much in vitro antagonism and in vivo disease resistance towards lactococcosis agents in aquaculture. Most in vitro studies with herbal extractives were performed against *L. garvieae* with no strong antibacterial activity, but essential oils, especially those that contain thymol or carvacrol, are more effective. The differences exhibited by the bacteriostatic and bactericidal functions for a specific extractive in different studies could be due to different bacterial strains or parts of chemotypes of the same plant. Despite essential oils being shown to have the best anti-*L. garvieae* activity in in vitro assays, the in vivo bioassays required further study. The extracts tested under in vivo conditions presented moderate efficacy, causing a decrease in mortality in infected animals, probably because they improved immune parameters before challenging tests. This review addressed the efficacy of medicinal herbs to lactococcosis and discussed the presented gaps.

## 1. Introduction

The aquaculture industry has seen a rapid acceleration in its development; as the most rapidly growing global agricultural sector, the aquaculture sector is now responsible for producing more than 50% of global seafood, with an average growth of 5.3% per year in the period 2001–2018 [1]. It is important to note that despite such rapid development, it is vitally important to pay attention to the processes used in order to maintain the quality standards of aquaculture products. Frequent worldwide outbreaks of infectious diseases, including bacterial diseases, are now one of major obstacles causing both huge financial losses and significant reductions in the quality standards of aquaculture production [2,3,4]. The initial reports of Gram-positive bacterial agents involved in systemic bacterial diseases in aquaculture species including rainbow trout (*Oncorhynchus mykiss*) came from Eastern Asian regions, and, since then, diseases caused by several different Gram-positive bacteria belonging to genera of *Lactococcus* [5,6], *Streptococcus* [7], *Vagococcus* [8], and *Carnobacterium* [9] have dramatically increased in global aquaculture [10,11,12].

The genus *Lactococcus*, which has the ability to produce lactic acid from glucose, consists of eleven species that can be divided in two phylogenetic groups: the first group contains *L. garvieae*, *Lactococcus lactis*, *Lactococcus taiwanensis*, *Lactococcus hircilactis*, *Lactococcus fujiensis*, and *Lactococcus formosensis*, and the second group comprises *Lactococcus laudensis*, *Lactococcus raffinolactis*, *Lactococcus chungangensis*, *Lactococcus plantarum,* and *Lactococcus piscium* [13]. Some strains, mainly *L. lactis*, are widely applied in industrial processes as starter cultures, probiotics and protective cultures [14,15,16]. *L. lactis* is generally recognized as safe by the US FDA and is suitable for the qualified presumption of safety approaches [17]. In recent years, attention has been paid to other species of *L. garvieae*, *L. raffinolactis*, *L. plantarum,* and *L. piscium* as potential pathogens of aquaculture species [18,19,20,21].

Among the species of *Lactococcus* genus, *L. garvieae* has been highlighted as one of the most serious global bacterial pathogens in the aquaculture sector, both in freshwater and marine fish, especially at water temperatures of >15 °C, but *L. lactis* and *L. piscium* seem to be limited to some highly valuable aquaculture species, such as salmonids and sturgeons, at various water temperatures [11,22,23]. Due to the widespread sources of the bacterial agents and disease spreading, as well as the heterogenicity of the bacterial stains implicated in the disease outbreaks, both vaccination and chemotherapy require more attention in future. The application of co-friendly environmental substances, such as medicinal herbs and probiotics, are nowadays a potential best alternative to antibiotic therapy and an immune enhancer against such bacterial diseases. This review addresses and discusses the efficacy of medicinal herbs and plants as means of prevention or for the protection of susceptible aquaculture species against diseases caused by *L. garvieae* and other *Lacococcus* species reported as the causative agents of lactococcosis in aquaculture.

## 2. Diseases Caused by *Lactococcus* Members in Aquaculture

### 2.1. Disease Caused by L. garvieae

**Figure 1:**
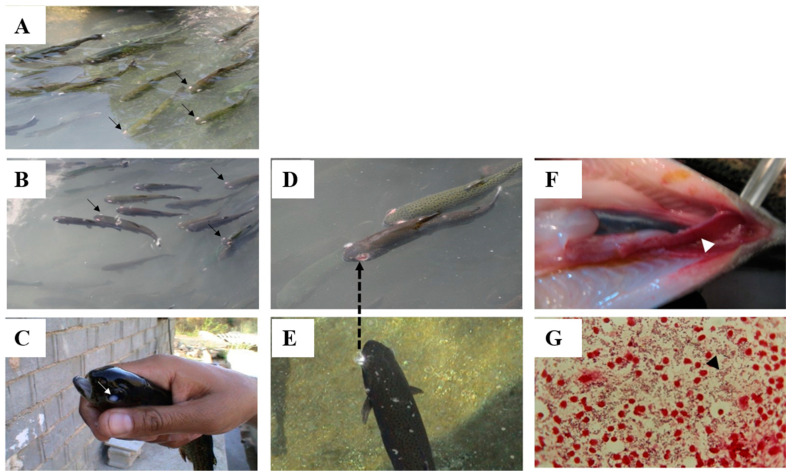
Rainbow trout growing in race ways and affected by *L. garvieae* showing: (**A**) typical bilateral exophthalmia and no change in the color skin, (**B**) typical bilateral exophthalmia and beginning of skin color change, (**C**) typical cataract and dark color, (**D**) bilateral exophthalmia and a complete loss of the eye, (**E**) darkening of body and an explosion of the eye, (**F**) hemorrhage of intestine, (**G**) direct Gram stain preparation of spleen of diseased fish showing huge numbers of Gram-positive coccoid cells confirmed as *L. garvieae* by phenotyping and molecular works. (photos by Professor Mehdi Soltani).

Lactococcosis, caused by *L. garvieae*, is a systemic hyperacute bacterial disease causing general hemorrhagic symptoms in susceptible aquatic organisms [24,25]. Based on their ability to agglutinate serum raised against *L. garvieae*, there are two groups of bacterial serotypes: non-agglutinating (KG−) and agglutinating (KG+) phenotypes [26]. The affected fish first become relatively anorexic, with a visible darkening of skin color, showing sluggish movement and abnormal behaviors, such as erratic and spiral swimming [11,24]. In the later stages of the disease the affected fish display signs of swollen abdomen, anal prolapsus, lateral or bilateral exophthalmia (Figure 1A,B), cataracts (Figure 1C), congestion of the internal organs, spleen and liver enlargement, accumulation of turbid ascitic fluid in the peritoneal cavity, and the presence of exudates in the brain [11,22,27,28]. Acute hyperemia and or extensive hemorrhage and petechiae of the organs, including the mucosal layers of the intestine, can also be seen in the diseased fish (Figure 1F), and in some cases the diseased fish show signs of explosion in the eyes prior to the loss of their eyes (Figure 1D,E) [11,22,29]. In advanced forms of the disease, a Gram stain preparation of hematopoietic tissue imprints, including spleen and kidney, can exhibit huge numbers of Gram-positive coccoid cells in single or chain forms (Figure 1G).

Pericarditis, peritonitis and meningitis, diffuse hemorrhage in the sclera of the eye, focal necrosis in the spleen and clumps of bacteria, hemorrhage in serosa of the swim bladder and in the interstitium of the skeletal muscles, degeneration and necrosis in epithelia of the stomach glands and their lumens full of necrotic material are among the identified histopathological findings in lactococcosis infection caused by *L. garvieae* [30,31,32]. Vascular changes in spleen and kidneys [33] and degenerations in the tubular epithelium with an increase in the melano-macrophage centers, hemorrhage in the form of a hematoma covering the myocardium and the bulbus arteriosa, petechial hemorrhage, vascular change, degeneration and necrosis are major histopathological findings. Lipid and ell infiltration in the liver, hemorrhage and vascular change in muscles, and petechial hemorrhage and edema in the gills are further microscopic changes reported in the infected fish by *L. garvieae* [31]. The severity of such pathological changes is, however, varied and depended on various factors, including level of virulent of bacterial strain, fish species and size and level of health management criteria, such as water temperature.

Evidence of bacterial cells in fish macrophage in tissues of spleen, kidney, heart (endothelial), and peritoneum are evidence of a septicemic condition, suggesting that macrophages play a key role in the host immune response to *L. garvieae* infection. However, intra-macrophage resistance of the bacteria can cause a spread of the pathogen to all tissues of fish by macrophages. Further, as in the young fish phagocytosis by macrophage activation may not be sufficient, thus, pathogenesis is a determinant factor, and the disease can progress.

Several factors play roles in the virulence of *L. garvieae*. Polysaccharide capsule is the major virulence factor in *L. garvieae* infection [29]. The capsulated strains resist to phagocytosis, but some non-capsulated strains are pathogenic in fish causing high mortality in rainbow trout [34], thus, the bacterial capsule may not the sole determinant of the bacterial pathogenicity. Haemolytic toxin is known to cause mortality in fish via intramuscular injection and an intracellular toxin with a low leukocidal activity reported by the bacterial isolates recovered from the diseased fish [35]. Plasmids of the virulent isolates contain a protein with an enzymatic domain corresponding to the family of actin-ADP-ribosyltransferases [36] that can kill eukaryotic cells by transferring ADP-ribose to essential proteins [37]. The toxicity of this protein in fish however, warranted future research works. The presence of a putative set of virulence factor genes (hly1, hly2, hly3, nox, sod, pavA, psaA), and proteins of enolase, lactate dehydrogenase phosphoenolpyruvate-protein phosphotransferase with roles in adhesion, cytolytic activity, oxidative stress tolerance, and metal homeostasis have been detected in strains of *L. garvieae*, including the avirulent reference strains ATCC^®^ 49156 and ATCC^®^ 4392, isolates from diseased rainbow trout in Turkey, France, Iran, Spain, and Italy [38], and fish pathogenic non-capsulated strains in South Africa [6]. These virulence lifestyle factors can indirectly contribute to host tissue damage through aiding in the infection process by evasion of the host’s innate immunity, systemic invasion, cofactor homeostasis, and spreading in the host and adhesion to host tissues. Further research works need to be directed studying the differential expression of virulence lifestyle and true virulence genes during growth in the host environment. Additionally, more studies need to assess the specific virulence factors responsible for the pathogenicity of *L. garvieae*, as putative virulence factor genes are present in both the fish pathogenic isolates and the avirulent isolates.

*L. garvieae* can invade a wide range of fish species both in warmwater, cold-water, freshwater and marine aquaculture species including Japanese eel (*Anguilla japonica*), Nile tilapia (*Oreochromis niloticus*), Red sea wrasse (*Coris aygula*), Pintado (*Pseudoplathystoma corruscans*), olive flounder (*Paralichthys olivaceous*), amberjack (*Seriola dumerili*) kingfish (*Seriola quinqueradiata*), rainbow trout (*Oncorhynchus mykiss*), grey mullet (*Mugil cephalus*), catfish (*Silurus glanis*), freshwater prawn (*Macrobrachium rosenbergii*), bottlenose dolphin (*Tursiops truncates*), and common octopus (*Octopus vulgaris*) worldwide [11,35,36,39,40,41,42,43,44]. The ability of the pathogen to adapt and survive in many environmental conditions is associated with its wide geographical distribution, it has been isolated from different aquatic and terrestrial animals [32,40,41,45,46], from rivers and sewage waters [47], and from different food and feedstuffs.

#### 2.1.1. Current Problems Associated with *L. garvieae* Infection

##### Frequent Re-Infection and Temporary Treatment

Several antibiotics, such as erythromycin, spiramycin, kitasamycin, josamycin (macrolide antibiotics), oxytetracycline, doxycycline, enrofloxacin, florfenicol, lincomycin, and amoxicillin, have been used to treat the disease caused by *L. garvieae* in fish farms [11,29,48,49]. One of problems with antibacterial agents is that they exhibit a well in vitro function towards *L. garvieae*, but their use in fish farms is not satisfied because of the loss of appetite of affected fish [50] or may be due to an ineffective metabolism of antibiotics in the diseased fish [3].

A following problem with chemotherapeutic treatment of lactococcosis in aquaculture is the increase in bacterial resistant [24,48], and multiple resistance to erythromycin, lincomycin, and oxytetracycline has been frequently reported in *L. garvieae* aquatic isolates [48,49,51,52] or to clindamycin, ampicillin and gentamicin in human isolates [53]. Development of R plasmids isolated from erythromycin-, lincomycin- and oxytetracycline-resistant *L. garvieae* isolates, macrolides, lincomycin, and tetracycline, or chloramphenicol have been reported [51,52]. The frequent re-infections of lactococcosis is also another limitation and constrain encountered with the disease in aquaculture, due to the formation of tissue granulation in different organs making the treatment become temporary [29,54]. In addition, a frequent treatment of diseased fish can cause an accumulation of antibiotics in the fish carcass raising a public health problem [55]. Further, the release of the drugs in the aquatic ecosystems can raise a development of bacterial resistance [56,57,58,59]. It is also very important to administrate the antibiotics of choice that is time-effective due to the fact that the time required for the bacterial isolation and antibiogram susceptibility tests as administration of unselective chemical compounds can increase the risk drug spreading in the aquatic environments. Thus, areas of the poor efficiency of chemotherapeutic agents under field conditions, the risks associated with the spread of antibiotic resistance determinants, and other methods of treatment or prevention are required further research studies. Prevention by vaccination is considered the best option to control lactococcosis, but there are still several limitations with the available vaccines, e.g., limitation of duration of immunization, required local or regional vaccines using different strains of the bacterium (autogenous vaccines), and no strong efficacy by bath vaccination compared to injection route [29,50,54,60,61].

##### *L. garvieae* as Zoonotic Disease

Disease caused by *L. garvieae* was first as the causative agent of subclinical mastitis in cattle, and is known as the infection agent in other ruminants and pneumonia in pigs [62,63]. Implication of *L. garvieae* in human clinical infections has been well demonstrated [63,64], and during recent years, several human infections by *L. garvieae* via handling and ingestion of raw fish have been reported [63], rising the status of the disease as an emerging zoonotic agent. The affected humans show endocarditis, bacteremia, hip prosthetic infection, cholecystitis, meningitis, urinary infection, espondilodiscitis, osteomyelitis, liver abscess, peritonitis, or bacterioascites [63,65,66,67]. In the case of human infective endocarditis, up to 2019, more than twenty-five cases of *L. garvieae* as the cause of infective endocarditis have been reported in the literature and compared to other Gram-positive cocci, *L. garvieae* affects more frequently patients (16%) with prosthetic valves compared to infective endocarditis (15.7%) caused by streptococcal members [67,68,69,70,71]. In a recent study by Malek et al. [71], infection by *L. garvieae* has been reported as an unusual cause of infective endocarditis in a 50-year-old male with mitral prosthetic valve and intracranial haemorrhage that was successfully treated using a combination of lindamycin, ancomycin, gentamicin, and ceftriaxone. The disease agent can be transmitted from aquatic products to human; thus, the role of foodborne transmission is still an important route of disease transmission to human [46,72] as some strains of *L. garvieae* with fish or shellfish source are responsible for human infections [73]. Studies demonstrating the host-specificity are essential to clarify the link between occurrence of the disease in aquaculture species and human infections especially in the regions where farmers are growing highly susceptible fish species, such as rainbow trout. For instance, from 64 fresh rainbow trout samples collected from fish market in Iran, 23.43% (15 samples) were positive for *L. garvieae* in their internal or muscles organs indicating the role of market contaminated fish in the transmission of disease to consumers [74]. Overall, despite its primary role as a bacterial fish pathogen [29], evidences of septicemia, endocarditis, and osteomyelitis caused by *L. garvieae* in humans raises the consumption of the infected fish as a major risk factor in humans, thus, in fish populations in which disease occurs, necessary attentions must be made prior to offering the fish for human consumption.

##### Economic Significant

Among the huge losses of production in aquaculture industry, the outbreaks by infectious diseases are the most serious problem that causes significant loss to aquaculture farmers, decrease food insecurity and incomes, and loss of job. It is reported that about 50% loss of aquaculture production is due to outbreaks by infectious diseases which are more severe in developing countries. The annual loss by infectious disease is up to USD 6 billion [75]. For example, about 15% of the total fish aquaculture production in China is lost due to disease outbreaks [76]. Such calculations are, however, rough estimation and, it is, therefore, crucial important to emphasize that there is not an actual annual estimation of economic losses caused by infectious diseases in global aquaculture. Even in the case of individual infectious disease, such as *L. garvieae,* there are no calculated data presenting an estimated loss in aquaculture sector. The economic impact by lactococcosis in farmed fish is, however, very high, especially in the regions where the susceptible species are cultured. For instance, based on our 20-year experience on farmed trout, the annual loss by the disease outbreaks in Iranian farmed trout is about USD 23 million that is associated with certain predisposing factors, such as poor health management and poor water quality, as well as growing of the susceptible species, rainbow trout, in this country. Even in developed countries, such as Japan, the economic losses caused by infectious diseases including lactococcosis was exceeded JPY 20 billion (USD 0.18 billion) prior to 1996 [77]. The annual estimation loss by *L. garvieae* in farmed fish is, therefore, a gap and required a necessary evaluation worldwide.

With new approaches in the biotechnology of vaccine production, there is a hope to control the diseases that impact the economics of the aquaculture sector. Thus, vaccination can be considered as the best choice to protect the fish farms from lactococcosis outbreaks, due to the poor efficiency of antimicrobial drugs in fish farms. Vaccination is a good tool to prevent the risks of spreading of antibiotic resistance factors [29]. However, use of autogenous vaccines against *L. garvieae* are more recommended due to their higher protection compared to non-autogenous vaccines [50]. Additionally, it is recommended to use such vaccines in combinations with the adjuvants/ or immuno-stimulators, e.g., probiotics, prebiotics, and medicinal herbs for ensuring a longer period of protection [29,78]. More details of different adjuvant formulations and immuno-stimulators are given by Vendrell et al. [29] and Soltani et al. [78]. It is, however, important to use a booster vaccination once an autogenous vaccine is in use because the efficacy of the available anti-*L. garvieae* vaccines is 5–6 months [78]. In addition to immunization, the frequency of lactococcosis outbreaks and level of morbidity and mortality can be reduced by improving the health management criteria and farm biosecurity, including enhancement of water quality parameters, especially water treatment; and avoiding overcrowding, overfeeding, and overhandling. As *L. garvieae* has a wide natural source including both warm-blooded and cold-blooded animals, disinfection of the water source used for aquaculture activity can significantly reduce the load of the bacterial agents in the water column.

### 2.2. Diseases Caused by Other Species of Lactococcus Genus

*L. lactis* strains are genetically classified into four subspecies of *lactis*, *cremoris*, *tractae,* and *hordniae* [13]. It is not a common veterinary pathogen, although it can cause cattle mastitis and be involved in septic arthritis of the neonatal calf. For example, several variants of *L. lactis* have been associated with bovine mastitis [79]. In humans, it has been reported as a cause of endocarditis, arthritis, and septicemia in patients, although this requires more clarification [80,81,82,83,84]. Up to date, there are only four reports of lactococcosis by *L. lactis* in an aquatic organisms. The first report was an outbreak of white tail disease in cultured giant freshwater prawn (*Macrobrachium rosenbergii*) in Taiwan [85]. The affected prawns were cloudy and whitish in the muscles, showing remarkable edema and necrosis and inflammation in the muscles and hepatopancreas. In subsequent report by Chen et al. [23] *L. lactis* subsp. *Lactis* was isolated from affected hybrid sturgeon, Bester (*Huso huso x Acipenser ruthenus*) with signs of anorexia, pale body color, reddish spots on the abdomen, enteritis, enlarged abdomen, rapid respiration rate ascites, and 70%–100% mortality. Microscopically, the affected sturgeons demonstrated extensive haemorrhagic multifocal necrotic foci of spleen and liver with degeneration of hepatic cells, lipid droplets and glycogen granules, necrosis and renal tubule epithelial swelling and hydropic degeneration in kidney, skin ulcers deep in underling muscles, appearance of present of immunocompetent cells in the stomach, and small focus on tips of gills and on the myocardium [23]. No histopathological changes were, however seen in the eyeball, cerebrum and meninges of affected fish. The third report was from silver carp (*Hypophthalmichthys molitrix*) with extensive skin lesions near the caudal peduncle and musculoskeletal lesion in the USA [86]. The fourth outbreak of infection by *L. lactis* has been reported as the cause of endocarditis valvularis, parientalis thromboticans in mature allis shad (*Alosa alosa*) in Europe in 2018 that could be associated with the stressors, such as capturing, transport, breeding, and low oxygen level [87]. Although, in some cases the disease was reproduced experimentally, the mechanisms of pathogenesis by *L. lactis* in aquatic animals warranted future research works.

The first and only report of *L. piscium* as the cause of disease in fish was by Williams et al. [88] who isolated the bacterium from affected rainbow trout named pseudo-kidney disease [24]. In our best knowledge, the direct involvement of the bacterium as a fish disease agent has never been evidenced due to no pathogenicity evidence for the isolated strains. The bacterium has also never again been isolated or identified by culture-independent techniques in the fish intestine microbiota.

The only report of *L. raffinolactis* infection in fish has been reported as a fish commensal or an opportunistic pathogen [89], but there are no further data regarding its pathogenic function in fish.

## 3. Phytotherapy of Lactococcosis in Aquaculture

### 3.1. In Vitro Studies

Almost all in vitro studies with vegetable and lichens extractives were performed against *L. garvieae*. For convenience, details of in vitro and in vivo studies have been included in Table 1 and Table 2. Overall, extracts do not show strong antibacterial activity against *L. garvieae*, but essential oils are more effective, mainly those that contain thymol or carvacrol. There are some differences on minimum inhibitory and bactericidal concentrations for the same extractive in different studies (Table 1). This may be due to the use of different bacterial strains or parts or chemotypes of the same plant. According to Ríos and Recio [90], for vegetable extractives in vitro experiments with concentrations higher than 1000 μg mL^−1^ for extracts or 100 μg mL^−1^ for isolated bioactive compounds should be avoided, and concentrations below 100 μg mL^−1^ for extracts and 10 μg mL^−1^ for isolated bioactive compounds can be considered very promising.

The aqueous, methanolic and ethanolic extracts of the aerial parts, fresh fruits, or leaves of 65 plants from Turkey showed weak or non-significant activity against *L. garvieae* obtained from Dr. Altınok, Sürmene Faculty of Marine Science, Karadeniz Technical University, Trabzon, Turkey by the disc diffusion method [91,92,93], and only the ethanolic extract of dwarf periwinkle (*Vinca minor*) presented moderate activity against this bacterium [92]. Experiments using the disc diffusion assay with the methanolic, acetone, and aqueous extracts of 13 lichen species demonstrated that only the methanolic and acetone extracts of oak moss (*Evernia prunastri*), cartilage lichens (*Ramalina farinacea* and *Ramalina fraxinea*) and beard lichen (*Usnea florida*), and acetone extract of lung lichen (*Lobaria pulmonaria*) demonstrated moderate activity against *L. garvieae* [94]. The dichloromethane, methanolic and ethyl acetate extracts of the aerial parts of Aster (*Jurinea humilis*) showed weak activity in vitro against *L. garvieae* A4 strain [95]. The ethanolic extracts of Persian violet (*Cyclamen pseudibericum*), hardy cyclamen (*C. hederifolium*), and Cilician cyclamen (*C. cilicium*) also show no significant activity by disk diffusion assay against *L. garvieae* recovered from diseased rainbow trout [96].

Using the same assay demonstrated that the essential oils of the flowers of lavender (*Lavandula angustifolia*), everlasting (*Helichrysum plicatum*), wormwood (*Artemisia absinthium*), leaves of pepper mint (*Mentha piperita*), sweet basil (*Ocimum basilicum*), wild marjoram (*Origanum majorana*), thyme (*Thymus vulgaris*), spiked thyme (*Thymbra spicata*), sage (*Salvia officinalis*), bay laurel (*Laurus nobilis*), lemon verbena (*Aloysia citriodora*), and seeds of black pepper (*Piper nigrum*) showed strong activity against *L. garvieae* strain provided by Dr. İlhan Altınok (Trabzon, Turkey), while the essential oils of flowers of French lavender (*Lavandula stoechas*), yarrow (*Achillea millefolium*), flower buds of clove (*Syzygium aromaticum*), leaves of rosemary (*Rosmarinus officinalis*), rose geranium (*Pelargonium graveolens*), parsley (*Petroselinum sativum*), river red gum (*Eucalyptus camaldulensis*), bark of cinnamon (*Cinnamomum verum*), roots of ginger (*Zingiber officinale*), and seeds of Chinese parsley (*Coriandrum sativum*) showed moderate activity, and the essential oils of leaves of common fennel (*Foeniculum vulgare*) and anise (*Pimpinella anisum*) presented weak activity [97]. Another experiment with this methodology confirmed the strong activity of the essential oil of thyme and the moderate activity of the essential oil of rosemary but stated that the essential oil of bay laurel exhibited a moderate activity against this bacterium [98]. The essential oil of oregano (*Origanum acutidens*) (main compound carvacrol) also showed strong activity against *L. garvieae* (ATCC 43921) [99]. However, the essential oils of ginger, black cumin (*Nigella sativa*), thyme and clove showed weak activity and that of watercress (*Eruca sativa*) did not present any activity against *L. lactis* [100]. The skin mucus of rainbow trout fed the ethanolic extract of stinging nettle (*Urtica dioica*) at 20 and 30 g kg diet^−1^ for 56 days inhibited the bacterial growth of *L. garvieae* in in vitro condition [101].

Under in vitro study we found that essential oil of Shirazi thyme (*Zataria multiflora*) (main compound: carvacrol) inhibits the capsule formation of *L. garvieae* isolates obtained from diseased rainbow trout because it suppresses the expression of the epsD capsule gene [102] and the expression of two other virulent factors, the Hly and PavA genes of the bacterium [10]. In the next study by a microdilution assay on 12 strains of *L. garvieae* isolated from diseased rainbow trout exhibited minimum inhibitory concentrations (MICs) ranging from 160 to 320 μL/mL by essential oils of either *Eucalyptus camaldulensis* or *Mentha pulegium* 60 min post incubation at 25°C, while no effect was found for *Aloe vera* essence, suggesting a potential use of *E. camaldulensis* and *M. pulegium* essences against lactococcosis caused by *L. garvieae* in farmed fish [103]. In addition, horse mint *(Mentha longifolia)* essential oil at 80 µL/mL was more inhibitory towards *L. garvieae* isolates recovered from diseased rainbow trout than lower concentrations [104]. Further, by a broth microdilution method, essential oils of *Rosmarinus officinalis* and *Cuminum cyminum* were inhibitory to 11 strains of *L. garvieae* recovered from diseased trout with identical MICs ranging from 0.12 to 1.0 µL/mL at 20°C, 30°C, and 37 °C [105]. More recently we demonstrated that *R. officinalis* essential oil (1,8-cineol and α-pinene were the predominant components) was significantly inhibitory to *L. garvieae* inoculated on the filet of trout at 0.135% for up to 15 days storage at 4°C and best inhibitory effect was seen when the essential oil was used in combination with nisin at 0.5 µL/mL [106].

It is difficult to compare the level of inhibitory activity of the extractives of medicinal herbs and plants with the antibiotics due to several factors involved including the mode of actions, type of assay and strains of bacterial tested. In their study by Maki et al. (2008) [107], 146 strains of *L. garvieae* isolated from diseased yellow tail (*Seriola quinqueradiata)* in Japan showed varying susceptibility to the 15 chemotherapeutic agents (Table 1), but no comparative data are available between the antibiotics and the extractives of medicinal herbs. All strains exhibited identical level of sensitivity to chloramphenicol, florfenicol and benzylpenicillin, and erythromycin and kanamycin, i.e., sensitive and moderately resistant, respectively, while in case of erythromycin, 98 strains were sensitive, two strains were moderately resistant and 46 strains were highly resistant. Against both lincomycin and tetracycline, 100 strains were sensitive and 46 strains were resistant, while all strains exhibited susceptibility to new generation of quinolones including ciprofloxacin enoxacin, floroxacin, norfloxacin, orbifloxacin, and ofloxacin but a low susceptibility to old generation, i.e., oxolinic acid. The possible reason for highly resistance of about 30% of the strains to erythromycin could be a frequent treatment of the disease by this antibiotic that has been used as one of the antibiotic of choice to lactococcosis in fish farmed.

### 3.2. In Vivo Studies

All in vivo studies were related to survival against *L. garvieae* infection, and, in most cases, the extractives of medicinal herbs and plants were added to the diets for various periods before the treated fish being challenged with *L. garvieae* infection. Overall, the essential oils that showed the best in vitro antibacterial activity against *L. garvieae* (Table 1) were not tested for the in vivo bioassays yet. The extractives tested under in vivo conditions presented moderate in vitro antibacterial activity against this bacterium or even were not tested in vitro. However, the dietary supplementation with all tested extractives reduced mortality of infected animals (Table 2), probably because they improved immune parameters before challenging the treated fish with *L. garvieae*. A 12-day feeding giant freshwater prawn (*Macrobrachium rosenbergii*) with hot-water extract of water hyacinth (*Eichhornia crassipes*) leaves at 1, 2, and 3 g kg^−1^ diet induced significantly higher survival rate after challenge with *L. garvieae* infection, but higher disease resistance was seen in the prawn treated with higher concentration of the extract [108]. In addition, the treated animals exhibited an enhancement in the immune responses including respiratory burst, phenoloxidase activity, superoxide dismutase activity, glutathione peroxidase, total hemocyte value, differential hemocyte count, transglutaminase activity, and phagocytic activity towards *L. garvtieae*. In the subsequent research work by Chang and Cheng [109], dietary addition of three tested water hyacinth extracts (Table 2) for 120 days increased survival and immune parameters, i.e., total hemocyte count, semi-granular and granular cells counts of giant freshwater prawn while phenoloxidase activity, respiratory bursts of hemocytes were not observed only with dietary addition of powder of this plant to the diet. Significantly more disease resistance to the pathogen was also obtained in the animals fed hot-water treated extracts, rather than dried powder, suggesting the suitability of hot-water extract as a better treatment strategy due to its efficacy and its availability and convenience. The leaves extract of water hyacinth contains various bioactive compounds including saponins, polyoses, and alkaloid salts known as the major substances with immunostimulant effect and antimicrobial activity [110]. The dietary addition of banana (*Musa acuminate*) peels aqueous extract at lower (1 g kg diet^−1^) or higher (6 g kg diet^−1^) concentrations for 120 days increased disease resistance of giant freshwater prawn towards *L. garvieae* infection [111] that could be in part due to an enhancement effect on the total hemocyte and granular cells populations, lipopolysaccharide, and β-1,3-glucan binding protein, transglutaminase, and crustin mRNA expression levels in hemocytes of treated giant freshwater prawn measured by the authors. Higher concentrations of the plant (3 and 6 g kg diet^−1^), however, presented higher survival that was correlated with a higher activity in the phenoloxidase and phagocytic levels [111]. In contrast, giant freshwater prawn fed lower dosage (0.6 g kg diet^−1^) of the aqueous extract of noni (*Morinda citrifolia*) for 63 days revealed higher disease resistance towards *L. garvieae* challenge than the animals treated with higher concentrations, i.e., 3 and 6 g kg diet^−1^ [112]. The potency of the plant on the animal immune responses exhibited that total hemocyte value, differential hemocyte count, respiratory burst of hemocytes, phenoloxidase, and transglutaminase activities, as well as the gene expressions of prophenoloxidase, transglutaminase, crustin, and lysozyme were more stimulated at the lower dosage than the higher ones [112]. These studies demonstrated that, as can be observed in the review of Elumalai et al. [113], the immunostimulant effects of herbal compounds and the optimum doses to trigger the best response are species specific and usually do not follow a dose-response relationship. Dietary addition of the oil seeds of argan (*Argania spinosa*) at 5, 10, and 20 mL kg diet^−1^ for 45 days increased the survival of Nile tilapia (*Oreochromis niloticus*) after challenge with *L. garvieae* infection compared with control fish [114]. At the higher argan oil seeds the survival of treated fish was, however reduced but was still higher than control group [114]. In all treatments with this oil, the value of immunocompetent cell size (leucocyte population), and activities of lysozyme and mieloperoxidase were enhanced pre- and post-challenged with *L. garvieae* [114] that could support the fish disease resistance to lactococcal infection. Apparently, the supplementation with argan oil improved the oil acid profile of the fish feed, which led to the immunomodulatory effects observed in Nile tilapia [114]. The dietary addition of the aqueous extract of Shiitake mushroom (*Lentinula edodes*) at 10 and 20 g kg diet^−1^ for 45 days enhanced rainbow trout survival challenged with *L. garvieae* infection [115]. This response may in part be due to an increase in the number of leucocytes, myeloperoxidase, lysozyme activity, phagocytic activity, and IgM measured in the treated fish [115]. Rainbow trout fed dietary supplementation with the aqueous extract of oyster mushroom (*Pleurotus ostreatus*) at 10 and 20 g kg diet^−1^ for 42 days demonstrated higher survival in challenge with *L. garvieae* infection that could be due to an enhancement in the phagocytic, lysozyme, and myeloperoxidase activities, as well as an increase in immunocompetent cell populations, i.e., neutrophils, monocytes, and total white blood cells [116]. This effect may be, at least in part, due to the presence of β-glucans in the oyster mushroom, as these compounds have immunomodulatory effect in fish [117]. The dietary addition of essential oil of oregano (*Origanum onites*) in rainbow trout diet at 3.0 mL kg diet^−1^ for 60 days avoided any mortality in fish post-challenge with *L. garvieae* infection [118]. Such a higher survival rate could be due to the stimulation effect of carvacrol that was the main compound (92.6%) of the essential oil as the treated fish demonstrated a higher level in the lysozyme activity than the control fish [118]. Eight-week feeding rainbow trout with stinging nettle (*Urtica dioica*) at 1%, 2%, and 3% demonstrated an improvement in the growth, immune responses and antagonistic activity against *L. garvieae* only in the fish treated with 2% or 3% of stinging nettle [101], indicating a dosage optimization is required before any treatment. The in vivo efficacy of the plant to *L. garvieae* infection is, however warranted further works. Feeding rainbow trout with tarragon (*Artemisia dracunculus*) methanolic extract (aerial parts) at 10, 20, and 30 g kg diet^−1^ for 56 days demonstrated an enhancement in the fish mucus bactericidal activity against *L. garvieae* [119], but data on the fish disease resistance against lactococcosis caused by *L. garvieae* warranted further works.

Another preventive treatment was the injection of the essential oils of black cumin (*Nigella sativa*) and savory (*Satureja bachtiarica*) both at 0.417, 0.833, or 1.667 mg kg fish^−1^ as adjuvant to *Streptococcus*/*Lactococcus* vaccine 42 days before infection of rainbow trout with *L. garvieae* [120]. However, the vaccinated fish did not show any significant change in relative survival percentage, antibody levels, and white blood cells number compared to vaccinated fish without any of the oils [120]. In a unique study by Bilen et al. [121] use of the methanolic extract of beard lichen (*Usnea barbata*) at 235, 470, and 705 mg kg fish^−1^ by gavage twice a day for 10 days exhibited higher survival than control fish that could be in part due to the enhancement in the immune related genes (IL-8, TGF-β, IL-12 Beta, TNFα1, IL-10, COX-2, IL-6, TLR5, C3, IGM, MHC-II, iNOS, IgT, IFN1, IFN2, and IFN reg) in the infected fish with *L. garvieae* [121]. The survival of treated fish at higher dosage, i.e., 705 mg kg^−1^ fish however, resulted in no significant difference compared with the control fish, but the survivals of positive controls (florfenicol and erythromycin each at 353 mg kg^−1^ fish) were significantly higher than all treated groups. The two lower doses of this extract also increased myeloperoxidase activity in the treated fish [121]. These effects may be, at least in part, due to one of its main compounds, usnic acid, which has immune and antibacterial effects [122]. Additional studies dealing with dietary supplementation with extractives of plants or compounds that improve immune parameters in aquatic animals (see reviews of [107,123,124]) to prevent against *L. garvieae*, *L. lactis* and infections are recommended.

Although, herbal medicinal products have been reported as a major source for discovering new pharmaceutical compounds that have been used to treat serious diseases, studies validating their toxicity are essential prior to be formulated and prescribed as the therapeutic agents. Unfortunately, minimum data are available concerning the toxicity and negative side effects of medicinal herb and plants in aquaculture species especially on commercial fish species. The lethal concentration (LC_50_) of *Eichhornia crassipes* in tilapia (*Sarotherodon melanotheron*) at 3 h of exposure was 16.37% of the effluent water of the plant [125], and *Morinda cutrifolia* at concentrations of 4.8 and 5.4 g/L, aqueous fruit extract caused a significant histopathological damage in tilapia gills [126]. In their study by Doleželová et al., 2011 [127] a 96 h LC_50_ value for *Syzygium aromaticum* clove oil in Zebra fish (*Danio rerio*) and guppy (*Poecilia* *reticulata*) were 18.2 ± 5.52 mg/L and 21.7 ± 0.8 mg/L, respectively. Administration of beard lichen at 5248 μg/L and 1252 μg/L caused abnormal development of larvae and impairs the growth of sea urchin (*Paracentrotus lividus)* and caused 50% mortality in copepod (*Tisbe battagliai)* at early life stages, respectively [128].

## 4. Conclusions

Disease outbreaks by *Lactococcus* species specially *L. garvieae* is one of the major concerns faced in the aquaculture production worldwide, and various biological and environmental variables, as well as the aquaculture practices and husbandry can affect the quantity and impacts of the morbidity and mortality. Data influencing the economic losses can, thus, assist to develop policies and strategies to reduce the losses by lactococcosis outbreaks in aquaculture industry. Lactococcosis outbreaks especially by *L. garvieae* are increasingly recognized as a significant and re-emerging bacterial disease in aquaculture, but there is no an estimation of its economic impacts. Data describing antagonistic activity and disease resistance efficacy of potential medicinal herbs and plants towards lactococcosis caused by *L. garvieae*, *L. lactis*, *L. piscium* and *L. raffinolactis* in finfish are not very much. Almost all in vitro studies with vegetable and lichens extractives were performed against *L. garvieae*. Despite no strong antibacterial activity by herb extracts against *L. garvieae*, essential oils especially those that contain thymol and carvacrol are more effective against *L. garvieae* strains. The exhibited differences on minimum inhibitory and bactericidal values for the same extractive in different studies could be due to the use of different bacterial strains or parts or chemotypes of the same plant. Despite best anti-*L. garvieae* activity by the essential oils under in vitro assays, the in vivo bioassays need be assessed yet. The extractives tested under in vivo conditions presented moderate antibacterial activity against this bacterium or even were not tested in vitro. However, the dietary supplementation with all tested extractives reduced mortality of infected animals, probably because they improved immune parameters before challenging the treated fish with *L. garvieae*.

## Figures and Tables

**Table 1:** Minimum inhibitory concentration (MIC) (μg mL^−1^ or μL mL^−1^) and minimum bactericidal concentration (MBC) (μg mL^−1^ or μL mL^−1^) of plant and lichen extractives against *Lactococcus garvieae*. The portion of the plant used to prepare the extractives were cited only if stated in the studies. Note: The MIC_50_ and MIC_90_ of 15 antibiotics against 146 strains of *L. garvieae* isolated from diseased fish are also given at the end of the table.

Origin/ Source of *L. garvieae*	Plant	Extractive	Major Compounds	MIC	MBC	Reference
Rainbow trout	*Camellia sinensis* (green tea-leaves)	Methanolic extract	Unknown	800	1100	Akbary, 2014
Rainbow trout	*Thymus vulgaris* (thyme), *Origanum vulgare* (oregano) and *Eucalyptus* sp.	Mix-oil^®^ (essential oils from leaves)	Citraconic anhydride, 1, 8-cineole, thymol	6.25	12.5	Amiri et al., 2020
Unknown	*Glycyrrhiza glabra* L. (black sugar-root)	n-hexane extract	Unknown	Unknown	5630	Asan-Ozusaglam et al., 2014
Unknown	*Glycyrrhiza glabra* L. (root)	Dichloromethane extract	Unknown	Unknown	1410	Asan-Ozusaglam et al., 2014
Type culture collection	*Lavandula angustifolia* (lavender)	Essential oil	Unknown	500	Unknown	Baba, 2020
Type culture collection	*Eugenia caryophyllus*	Essential oil	Unknown	250	Unknown	Baba, 2020
Rainbow trout	*Mentha piperitae* (pepper mint)	Essential oil	Unknown	500	Unknown	Baba, 2020
Rainbow trout	*Rosmarinus officinalis* (rosemary)	Essential oil	Unknown	500	Unknown	Baba, 2020
Rainbow trout	*Cinnamomum zeylanicum* (cinnamon)	Essential oil	Unknown	250	Unknown	Baba, 2020
Rainbow trout	*Nigella sativa* (black cumin)	Essential oil	Unknown	250	Unknown	Baba, 2020
Strain O41	*Lavandula officinalis* (true lavender-flowers)	Ethanolic extract	Essential oil (linalyl acetate), tannins, coumarins, flavonoids, and phytosterols	4200	8400	Bulfon et al., 2014
Strain O41	*Melissa officinalis* (lemon balm-leaves)	Ethanolic extract	Rosmarinic acid, essential oil (citral, citronellal, β-caryophyllen), caffeic acid, and chlorogenic acidderivatives	8400	33,600	Bulfon et al., 2014
Strain O41	*Ocimum basilicum* (sweet basil-flowering plant)	Ethanolic extract	Essential oil (linalool, estragol, camphor, eugenol, ocimene, cineol, sesquiterpenes), tannins, favonoids, caffeic acid, and esculoside	16,800	Unknown	Bulfon et al., 2014
Strain O41	*Origanum vulgare* (oregano-inflorescence)	Ethanolic extract	Carvacrol, thymol, γ-terpinene, p-cymene, limonene, linolool, and borneol	4200	33,600	Bulfon et al., 2014
Strain O41	*Orthosiphon stamineus* (Java tea-leaves)	Ethanolic extract	Essential oil (sesquiterpenes), flavones, triterpenoid, saponins, vitamins, and organic salts	33,600	Unknown	Bulfon et al., 2014
Strain O41	*Rosmarinus officinalis* (leaves)	Ethanolic extract	Essential oil (eucalyptol, α-pinene, camphor, borneol), flavonoids, rosmarinic acid, and terpenes	8400	Unknown	Bulfon et al., 2014
Strain O41	*Salvia officinalis* (sage-leaves)	Ethanolic extract	Essential oil (thujone, monoterpenes, and sesquiterpenes), tannins, bitter substances, and flavonoids	4200	33,600	Bulfon et al., 2014
Strain O41	*Thymus vulgaris* (leaves)	Ethanolic extract	Essential oil (thymol, carvacrol, p-cimol, and terpinene), tannins, flavonoids and triterpenes	Unknown	Unknown	Bulfon et al., 2014
Strain O41	*Vaccinium vitis-idaea* (lingonberry-leaves)	Ethanolic extract	Phenolic glycosides (arbutin and hydroquinone), tannins, flavonoids (iperoside, avicularin, isoquercitrin), terpenic acids (ursolic and oleanolic acids), organic acids, and mineral salts	4200	Unknown	Bulfon et al., 2014
Rainbow trout	*Glycyrrhiza glabra* L. (root)	Ethanolic extract	Unknown	920	Unknown	Fereidouni et al., 2013
Rainbow trout	*Peganum harmala* (wild rue-seed)	Methanolic extrac	Unknown	105	Unknown	Fereidouni et al., 2013
Rainbow trout	*Trachyspermum copticum* (carum ajowan-seed)	Ethanolic extract	Unknown	453	Unknown	Fereidouni et al., 2013
Rainbow trout	*Myrtus communis* (myrtle-leaves)	Essential oil	Unknown	672	Unknown	Fereidouni et al., 2013
Rainbow trout	*Juglans regia* (English walnut-leaves)	Ethanolic extract	Unknown	510	Unknown	Fereidouni et al., 2013
Rainbow trout	*Quercus branti* Lindley (Brant’s oak-seed)	Ethanolic extract	Unknown	978	Unknown	Fereidouni et al., 2013
Rainbow trout	*Tanacetum parthenium* (feverfew-leaves)	Essential oil	Unknown	824	Unknown	Fereidouni et al., 2013
Rainbow trout	*Satureja bachtiarica* Bung. (savory-leaves)	Essential oil	Unknown	126	Unknown	Fereidouni et al., 2013
Rainbow trout	*Glycyrrhiza glabra* L.(root)	Ethanolic extract	Glycyrrhizinic acid	>1000	>1000	Goudarzi et al., 2011
Rainbow trout	*Satureja bachtiarica* Bung. (aerial parts)	Essential oil	Phenols: Carvacrol, thymol	8	16	Goudarzi et al., 2011
Rainbow trout	*Satureja bachtiarica* Bung. (aerial parts)	Ethanolic extract	Phenols: Carvacrol, thymol	>1000	>1000	Goudarzi et al., 2011
Rainbow trout	*Punica granatum* (pomegranate-flowers)	Ethanolic extract	Polyphenols: pomegranatate	>1000	>1000	Goudarzi et al., 2011
Rainbow trout	*Quercus branti* Lindley (seed/flour)	Ethanolic extract	Tannins	>1000	>1000	Goudarzi et al., 2011
Rainbow trout	*Echinophora platyloba* DC. (prickly parsnip-aerial parts)	Essential oil	Monoterpenes: trans-β-ocimene	>1000	>1000	Goudarzi et al., 2011
Rainbow trout	*Echinophora platyloba* DC. (aerial parts)	Ethanolic extract	Monoterpenes: trans-β-ocimene	>1000	>1000	Goudarzi et al., 2011
Rainbow trout	*Heracleum lasiopetalum* Boiss. (fruits)	Ethanolic extract	Sesquiterpene hydrocarbons: Germacrene-D	>1000	>1000	Goudarzi et al., 2011
Rainbow trout	*Kelussia odoratissima* Mozaff. (wild celery-leaves)	Ethanolic extract	Z-ligustilide	>1000	>1000	Goudarzi et al., 2011
Rainbow trout	*Stachys lavandulifolia* Vahl (wood betony-flowers)	Ethanolic extract	Sabinene, α-pinene, β-myrcene	>1000	>1000	Goudarzi et al., 2011
Rainbow trout	*Thymus daenensis* Celak. (common thyme-aerial part/inflorescence)	Ethanolic extract	Phenols: thymol, carvacrol	>1000	>1000	Goudarzi et al., 2011
Rainbow trout	*Thymus daenensis* Celak. (Aerial part/inflorescence)	Essential oil	Phenols: thymol, carvacrol	8	16	Goudarzi et al., 2011
Rainbow trout	*Myrtus communis* (leaves)	Ethanolic extract	α-pinene, 1,8-cineole, myrtenyl acetate	>250	>500	Goudarzi et al., 2011
Rainbow trout	*Myrtus communis* (leaves)	Essential oil	α-pinene, 1,8-cineole, myrtenyl acetate	>1000	>1000	Goudarzi et al., 2011
Rainbow trout	*Thymbra spicata* (spiked thyme-aerial part/inflorescence)	Essential oil	Phenols: thymol, carvacrol	8	16	Goudarzi et al., 2011
Rainbow trout	*Bunium persicum* (Boiss.) K.- Pol. (black caraway-fruits)	Essential oil	γ-terpinen-7-al, cuminaldehyde, γ terpinene	8	16	Goudarzi et al., 2011
Rainbow trout	*Teucrium polium* (felty germander-aerial parts)	Essential oil	α-pinene, linalool	>1000	>1000	Goudarzi et al., 2011
Rainbow trout	*Alhagi maurorum* (camelthorn-aerial parts)	Essential oil	Alhagidin, alhagitin, quercetin, catechin	>1000	>1000	Goudarzi et al., 2011
Rainbow trout	*Zataria multifora* Boiss. (Shirazi thyme)	Essential oil	Phenols: thymol, carvacrol	4	8	Goudarzi et al., 2011
Olive flounder (*P. olivaceus*)	*Zingiber officinale* (ginger)	Essential oil		2000	2000	Hossain et al., 2019
*L. garvieae* GQ850376	*Eucalyptus globulus* (southern blue gum-aerial parts)	Essential oil	1,8-eucalypol, pinene, terpineol acetae, globulol	250	250	Mahmoodi et al., 2012
*L. garvieae* GQ850376	*Eucalyptus globulus* (aerial parts)	Methanolic extract	1,8-eucalypol, pinene, terpineol acetae, globulol	500	500	Mahmoodi et al., 2012
*L. garvieae* GQ850376	*Zataria multiflora* (aerial parts)	Essential oil	phenolic monoterpene, Carvacrol, alpha-pinene	7.8	15.6	Mahmoodi et al., 2012
*L. garvieae* GQ850376	*Zataria multiflora* (aerial parts)	Methanolic extract	phenolic monoterpene Carvacrol, alpha-pinene	15.6	15.6	Mahmoodi et al., 2012
*L. garvieae* GQ850376	*Anethum graveolens* (dill-seed)	Essential oil	D-carvacrol, limonene, dill apiole, E-dihydrocarvone, Z-dihydrocarvone	62.4	125	Mahmoodi et al., 2012
*L. garvieae* GQ850376	*Anethum graveolens* (seed)	Methanolic extract	D-carvacrol, limonene, dill apiole, E-dihydrocarvone, Z-dihydrocarvone	125	125	Mahmoodi et al., 2012
*L. garvieae* GQ850376	*Rosmarinus officinalis*	Essential oil	1,8-cineole, alpha-pinene, toluene	15.6	31.2	Mahmoodi et al., 2012
*L. garvieae* GQ850376	*Rosmarinus officinalis*	Methanolic extract	1,8-cineole, alpha-pinene, toluene	31.2	31.2	Mahmoodi et al., 2012
Rainbow trout	*Citrus paradisi* (grapefruit), *Citrus reticulata* (tangerine), *Citrus aurantium* ssp. *bergamia* (bergamot), *Citrus sinensis* (sweet orange)	Biocitro ^®^ (blend of citrus extracts)	Ascorbic acid, citrus bioflavonoids (hesperidin, naringin, quercetin, rutin) and organic acids	2.0	Unknown	Mora-Sánchez et al., 2020
Tilapia (*O. andersonii*)	*Capsicum annum* (Chili pepper)	Methanolic extract (capsaicin)	Unknown	Unknown	196.7	Ndashe et al., 2020
Olive flounder	*Citrus aurantifolia* (key lime-peel)	Essential oil	Limonene, γ-terpinene, β-pinene	0.125% (*v*/*v*)	1% (*v*/*v*)	Pathirana et al., 2018
Olive flounder	Limonene	Commercial trans-limonene (>99%)	Limonene	0.031% (*v*/*v*)	0.025% (*v*/*v*)	Pathirana et al., 2018
Olive flounder	*Syzygium aromaticum* (clove-buds)	Essential oil	Eugenol, β-caryophyllene, α-humulen, eugenyl-acetate	0.5% (*v*/*v*)	1% (*v*/*v*)	Pathirana et al., 2019a
Olive flounder	Commercial eugenol (>99%)	Isolated compound eugenol	Eugenol	1% (*v*/*v*)	1% (*v*/*v*)	Pathirana et al., 2019a
Olive flounder	*Cinnamomum zeylanicum*	Essential oil	Cinnamaldehyde, eugenol, β-Caryophyllene	0.015% (*v*/*v*)	0.031% (*v*/*v*)	Pathirana et al., 2019b
Olive flounder	Commercial trans-cinnamaldehyde (>99%) (Sigma-Aldrich)	cinnamaldehyde	Cinnamaldehyde	0.003% (*v*/*v*)	0.015% (*v*/*v*)	Pathirana et al., 2019b
Rainbow trout	*Argania spinose* L. (argan-oil)	Essential oil	Oleic acid, linoleic acid, palmitic acid, stearic acid	250	Unknown	Öntas et al., 2016
Rainbow trout	*Citrus limon L.* (lemon-peel)	Essential oil	Limonene, γ-terpinene, β-pinene, α-terpineol, myrecene and terpinolene	500	Unknown	Öntas et al., 2016
Strain ATCC43921	*Cinnamomum verum* (cinnamon-bark)	Essential oil	Unknown	120	Unknown	Rattanachaikunsopon et al., 2009
Strain ATCC43921	*Ocimum sanctum* (holy basil-leaves)	Essential oil	Unknown	240	Unknown	Rattanachaikunsopon et al., 2009
Strain ATCC43921	*Zingiber officinale* (roots)	Essential oil	Unknown	120	Unknown	Rattanachaikunsopon et al. 2009
Strain ATCC43921	*Syzygium aromaticum* (flower buds)	Essential oil	Unknown	30	Unknown	Rattanachaikunsopon et al., 2009
Rainbow trout	*Zataria multiflora* (aerial parts)	Essential oil	Carvacrol, benzene and phenol	0.12	0.12	Soltani et al., 2014
Rainbow trout	*Allium sativum* (garlic-edible parts)	Essential oil	trisulfide, di-2-propenyl, disulfide, di-2-propenyl and trisulfide, methyl 2-propenyl	0.5	1	Soltani et al., 2014
Rainbow trout	*Cinnamomum zeylanicum* (bark)	Essential oil	cinnamic aldehyde, linalool, ortho methoxy cinnamic aldehyde and 1,8-cineole	0.5	0.5	Soltani et al., 2014
*S. quinqueradiata*	Chloramphenicol			0.8 ^a^	1.6 ^b^	Maki et al., 2008
*S. quinqueradiata*	Ciprofloxacin			1.6 ^a^	3.13 ^b^	Maki et al., 2008
*S. quinqueradiata*	Erythromycin			0.1 ^a^	800 ^b^	Maki et al., 2008
*S. quinqueradiata*	Enoxacin			6.25 ^a^	12.5 ^b^	Maki et al., 2008
*S. quinqueradiata*	Florfenicol			1.6 ^a^	1.6 ^b^	Maki et al., 2008
*S. quinqueradiata*	Floroxacin			12.5 ^a^	12.5 ^b^	Maki et al., 2008
*S. quinqueradiata*	Kanamycin			25 ^a^	50 ^b^	Maki et al., 2008
*S. quinqueradiata*	Lincomycin			25 ^a^	800 ^b^	Maki et al., 2008
*S. quinqueradiata*	Norfloxacin			6.25 ^a^	12.5 ^b^	Maki et al., 2008
*S. quinqueradiata*	Oxolinic acid			400 ^a^	800 ^b^	Maki et al., 2008
*S. quinqueradiata*	Orbifloxacin			1.6 ^a^	1.6 ^b^	Maki et al., 2008
*S. quinqueradiata*	Ofloxacin			3.13 ^a^	6.25 ^b^	Maki et al., 2008
*S. quinqueradiata*	Penzylpenicillin			0.8 ^a^	1.6 ^b^	Maki et al., 2008
*S. quinqueradiata*	Streptomycin			25 ^a^	50 ^b^	Maki et al., 2008
*S. quinqueradiata*	Tetracycline			12.5 ^a^	400 ^b^	Maki et al., 2008

Letters a and b showing MIC_50_ and MIC_90_, respectively.

**Table 2:** Efficacy of medicinal herbs and plants on the survival of aquatic animals infected with *Lactococcus garvieae*. The portion of the plant used to prepare the extractives were cited only if stated in the studies.

Host	Plant	Extractive	Dosage/Duration	Survival Increase Compared to Control (%) ^2^	Reference
Giant freshwater prawn (*Macrobrachium* *rosenbergii*)	*Eichhornia crassipes* (water hyacinth-leaves)	Hot-water extract	1 g kg diet^−1^, 12 days 2 g kg diet^−1^, 12 days 3 g kg diet^−1^, 12 days	↑57.3 ↑128.6 ↑171.4	Chang et al., 2013
Giant freshwater prawn (*M. rosenbergii*)	*Eichhornia crassipes* (leaves)	Powder	20 g kg diet^−1^, 120 days	↑44.3	Chang et al., 2016
		Hot-water extract	20 g kg diet^−1^, 120 days	↑89.0	
		Aqueous extract ^1^	2 g kg diet^−1^, 120 days	↑89.0	
		Dreg of aqueous extract ^1^	18 g kg diet^−1^, 120 days	↑77.7	
Giant freshwater prawn (*M. rosenbergii*)	*Musa acuminate* (banana-peel)	Aqueous extract	1 g kg diet^−1^, 120 days 3 g kg diet^−1^, 120 days 6 g kg diet^−1^, 120 days	↑200 ↑300 ↑467	Rattanavichai et al., 2015
Giant freshwater prawn (*M. rosenbergii*)	*Morinda cutrifolia* (noni)	Aqueous extract	0.6 g kg diet^−1^, 21 days 3 g kg diet^−1^, 21 days 6 g kg diet^−1^, 21 days	↑250 ↑50.4 NS	Halim et al., 2017
Nile tilapia (*Oreochromis niloticus*)	*Argania spinosa* (argan-seeds)	Oil	5 mL kg diet^−1^, 45 days 10 mL kg diet^−1^, 45 days 20 mL kg diet^−1^, 45 days	↑66.7 ↑91.7 ↑86.1	Baba et al., 2017
Rainbow trout (*Oncorhynchus mykiss*)	*Lentinula edodes* (Shiitake mushroom)	Aqueous extract	10 g kg diet^−1^, 45 days 20 g kg diet^−1^, 45 days	↑79.0 ↑109.7	Baba et al., 2015
Rainbow trout (*O. mykiss*)	*Pleurotus ostreatus* (oyster mushroom)	Aqueous extract	10 g kg diet^−1^, 42 days 20 g kg diet^−1^, 42 days	↑40.0 ↑60.1	Uluköy et al., 2016
Rainbow trout (*O. mykiss*)	*Usnea barbata* (beard lichen)	Methanolic extract	230 mg kg fish^−1 (a)^ 460 mg kg fish^−1 (a)^ 690 mg kg fish^−1 (a)^	↑62.4 ↑45.3 NS	Bilen et al., 2019
Three spotted tilapia (*Oreochromis andersonii*)	Capsaicin	Isolated compound	1.97 mg kg fish^−1 (b)^	80% survival vs. 0% survival in control	Ndashe et al., 2020
Rainbow trout (*O. mykiss*)	*Origanum onites* (oregano)	Essential oil	0.125 mL kg diet^−1^, 56 days 1.5 mL kg diet^−1^, 56 days 2.5 mL kg diet^−1^, 56 days 3.0 mL kg diet^−1^, 56 days	↑54 ↑92 ↑84 No mortality	Diler et al. 2017
Nile tilapia (*Oreochromis niloticus*)	*Syzygium aromaticum* (clove-buds)	Essential oil	5 mL kg diet^−1^, 5 days 10 mL kg diet^−1^, 5 days 20 mL kg diet^−1^, 5 days 30 mL kg diet^−1^, 5 days	↑40 ↑70 ↑80 No mortality	Rattanachaikunsopon et al., 2009
Mullet (*Mugil cephalus*)	* TCM	Aqueous extract of the powder	5 g kg fish^−1^, 28 days 10 g kg fish^−1^, 28 days 20 g kg fish^−1^, 28 days	NS ↑230.8 ↑184.6	Choi et al., 2014
Rainbow trout (*O. mykiss*)	*Citrus paradisi* (grapefruit), *Citrus reticulata* (tangerine), *Citrus aurantium* ssp. *bergamia* (bergamot), *Citrus sinensis* (sweet orange)	Biocitro ^®^ (blend of these extracts)	0.75 g kg diet^−1^, 28 days	↑120	Mora-Sánchez et al., 2020

^1^ Aqueous extract was the supernatant of the hot-water extract filtered and centrifuged. The left filtered product and sediment were the dreg of aqueous extract. ^2^ survival increase calculated by the equation: (survival treatment × 100)/survival control–100. NS = nonsignificant difference from control. * TCM = traditional Chinese medicine: composed by the plants *Rhizoma coptidis*, *Radix scutellariae*, *Cortex phellodendri*, *Fructus gardeniae jasminoidis*, *Fructus forsythiae*, and *Flos lonicerae japonicae*. (^a^) = Gavage in infected fish twice a day for 10 days. (^b^) = Injected in the same day of infection with the bacteria.

## Data Availability

The study did not report any data.
